# Plasticity of female reproductive resource allocation depends on the presence or absence of prior environmental sex determination in *Ceratopteris richardii*

**DOI:** 10.1002/ece3.4159

**Published:** 2018-05-20

**Authors:** Taylor T. Goodnoe, Jeffrey P. Hill

**Affiliations:** ^1^ Department of Biological Sciences Idaho State University Pocatello Idaho

**Keywords:** allometry, labile sex expression, phenotypic plasticity, reproductive output

## Abstract

Resource allocation plasticity enables individuals to alter patterns of nutrient use between reproductive and vegetative output to better fit their current environment. In sexually labile plant species, abiotic environmental factors can influence expression of dimorphic gender, resulting in environmental sex determination (ESD), which potentially reduces the need for plasticity of resource allocation by preemptively matching an individual’s future nutrient demands to resource availability in its location. *Ceratopteris richardii* gametophytes exhibit gender‐dependent differences in relative carbon and nitrogen content, and ESD in certain nutrient environments. This study examined whether prior ESD in *C. richardii* gametophyte populations reduced subsequent plasticity of reproductive allocation compared to instances where no ESD occurred, by quantifying phenotypic responses to reduced P, N, or CO_2_ availabilities. All three nutrient‐limited environments resulted in decreased size of egg‐bearing (meristic) gametophytes compared to nonlimited environments, but gametophytes failed to respond to N and CO_2_ limitation at the time of sex determination, resulting in no ESD. N limitation resulted in a predictable allometric re‐allocation of resources based on small gametophyte size, whereas CO_2_ limitation caused a change in reproductive output consistent with true plasticity. Withholding exogenous P caused ESD and had no effect on relative reproductive output of resultant meristic gametophytes because the size decrease was minor. Under P limitation, ESD matched the resource demands of gender phenotypes to their environment before the onset of developmental dimorphism, reducing the need for large allocation adjustments after sex determination.

## 1 INTRODUCTION

Sessile plants cannot escape unfavorable nutrient environments, but phenotypic plasticity, strict genetic adaptation, or most commonly a combination of both can enable growth and development across a wide range of edaphic conditions (Bradshaw, 1965; Dorken & Barrett, 2004; Nunney, 2016; Weiner, 2004). To persist in a heterogeneous or changing environment, individual plants adjust allocation of resources to different structures based on local abiotic factors, including resource availability (DeBiasse & Kelly, 2016; Weiner, 2004). Environmentally responsive phenotypes play a vital role in an organism’s life history, and their underlying ontogenies are shaped by natural selection to optimize fecundity and overall individual fitness (Bazzaz & Grace, 1997; Bennett, Roberts, & Wagstaff, 2011).

Plant responses to environmental variation and potential limitation of necessary nutrients require the strategic allocation of resources based on trade‐offs between important functions. For example, sex allocation theory addresses the plasticity of resource partitioning to male and female functions in hermaphrodites (Charlesworth & Charlesworth, 1981; Delph, 2003). Additionally, strategic allocation of biomass to any reproductive structures, regardless of gender, must be balanced in relation to allocation to vegetative structures (Mandák & Pyšek, 1999; Reekie & Avila‐Sakar, 2005; Reekie & Bazzaz, 1987).

Before reproduction can begin in the plant life cycle, individuals must grow to a specific minimum size, confirming there are costs and trade‐offs between growth and reproduction (Obeso, 2002; Reekie & Bazzaz, 1987; Reznick, 1985; Roff, 1992; Van Noordwijk & de Jong, 1986). Because reproduction expends nutrients provided by vegetative tissues (Delerue, Gonzalez, Atlan, Pellerin, & Augusto, 2013), a negative correlation between vegetative growth and the development of reproductive structures results. When resource availability is limited, excessive reproductive allocation will impede vegetative growth; overinvestment in sexual functions in the absence of adequate supporting vegetative biomass risks reproductive failure and may ultimately prove detrimental to individual fitness (Obeso, 2002).

Plants frequently have characteristic species‐specific relationships between reproductive output (R) and vegetative biomass (V; i.e., an R‐to‐V relationship, hereafter termed “R‐V”), even when nutrients are not limiting (Delerue et al., 2013; Klinkhamer, Meelis, De Jong, & Weiner, 1992). When individuals of the same species are grown in different patches of a heterogeneous environment, they may exhibit phenotypic differences in growth and reproduction that depend mainly on differences in size, an allometric response. True plasticity of resource allocation due to nutrient limitation implies a change in the R‐V relationship that is not simply due to a change in growth rate or overall size (Delerue et al., 2013; Weiner, 2004). An important focus of this study is to determine whether nutrient limitation evokes true R‐V plasticity in a species with an intriguing life history feature that has a direct bearing on resource allocation strategies: labile sex expression.

Labile sex expression—sex determination plasticity—occurs in plants species where sex is not genetically determined, and provides a striking opportunity for environmental factors to influence nutrient allocation strategies by partitioning individuals within a population into separate sexes before sexual development even begins (Korpelainen, 1998). The ability to regulate sex based on environmental conditions is considered adaptive when the environment is heterogeneous with respect to key abiotic factors and sexually undetermined individuals in a population randomly disperse to specific habitat patches (Bull, 1983; Charnov & Bull, 1977; Janzen & Phillips, 2006). In a resource‐limited environment—where an individual can either become a below average female or an above average male—developmental constraints arising from disparities in future nutrient demands between genders could be diminished by environmental sex determination (ESD), which shifts the population sex ratio to allow better phenotypic alignment with local conditions. Nutrient deficient patches are expected to yield populations with a higher probability of more individuals becoming male because the likelihood of genetic transmission through male gametes is higher (Charnov & Bull, 1977).

**Figure 1:**
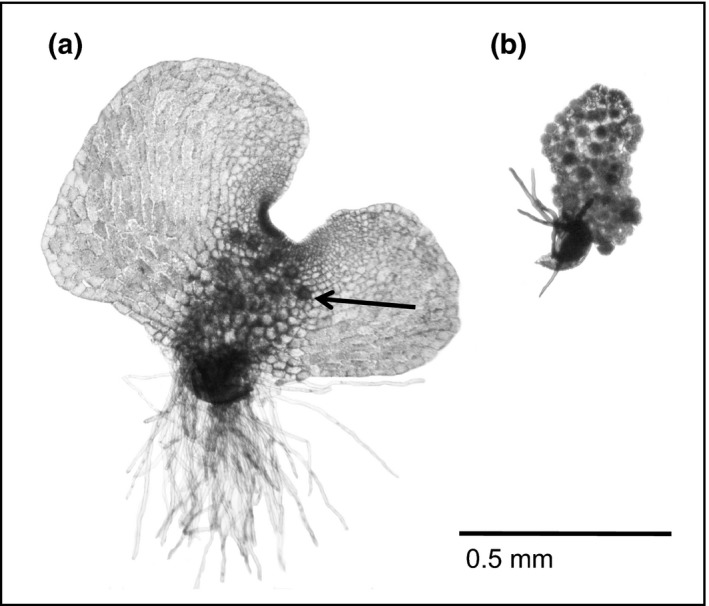
Representative meristic female (a) and ameristic male (b) gametophytes of *Ceratopteris richardii*. Archegonia (i.e., female gametangia, each containing a single egg) are visible around the lateral meristem of the meristic gametophyte (e.g., arrow). Most cells of the male gametophyte have differentiated into an antheridium (i.e., male gametangium, each containing sperm). Meristic gametophytes can also become hermaphroditic by developing several antheridia around the perimeter of the gametophyte tissue, away from the meristem. Ameristic gametophytes are always strictly male

In the pteridophyte species *Ceratopteris richardii* (Figure 1), gametophyte sex determination is labile (Banks, 1999; Scott & Hickok, 1987). Macronutrients have recently been shown under certain CO_2_ and glucose regimes to influence the probability of meristem development (Goodnoe, Hill, & Aho, 2016). Meristems are the site of female gametangial development (Figure 1a) and thus a prerequisite for expression of female (“meristic”) sexual functions (Hickok, Warne, & Slocum, 1987; Tanurdzic & Banks, 2004). Gametophytes lacking meristem formation (“ameristic”) develop as males (Figure 1b). These dimorphic *C. richardii* gametophyte phenotypes also differ in their nutrient demands; per unit plant dry mass, meristic gametophytes contain relatively more nitrogen (N) and less carbon (C) than ameristic gametophytes. Thus, N and C demands reliably differ by sex (Goodnoe & Hill, 2016). That suggested it might be possible to turn ESD on or off in vitro based on the nutrient context in which gametophytes are grown, in a species where future nutrient demands depend predictably on sex expression.

According to Liebig’s law of the minimum, organisms feeding on a finite pool of required nutrients will become limited by whichever resource is least abundant compared to their needs (Hiddink & Kaiser, 2005; Liebig, 1842; Sterner & Elser, 2002). By imposing Liebig limitation via variation or elimination of N and P concentrations, and variation in CO_2_—nutrients that are required in specific concentrations and stoichiometries for growth and development by all organisms—we created environments where ESD was present and environments where it was absent. Because plasticity of allocation may be influenced by the extent to which an individual’s gender was preemptively matched to the local environment, we studied female reproductive output in relation to meristic plant size to evaluate resource allocation strategies of meristic *C. richardii* gametophytes once gender had been determined, in settings with and without prior ESD. By inducing nutrient limitation, changes in R were evaluated expressly as a function of experimental decreases in allocation to V.

Although a negative relationship between reproductive and vegetative allocation is theoretically inescapable (Obeso, 2002), demonstrating that relationship has previously proven difficult because the collective noise of multiple allocation strategies operating concurrently to regulate the development of numerous organs often confounds the signal of interest (Metcalf, 2016; Van Noordwijk & de Jong, 1986). The relatively simple morphology of gametophytes (Figure 1) compared to flowering plant sporophytes helped mitigate those issues in this study system. To eliminate genetic variance as another confounding source of phenotypic variation, plasticity of resource allocation (sensu Hendry, 2016) was assessed in genetically identical individuals from an inbred line (Hickok, Warne, & Fribourg, 1995) cultured under different nutrient conditions.

**Figure 2:**
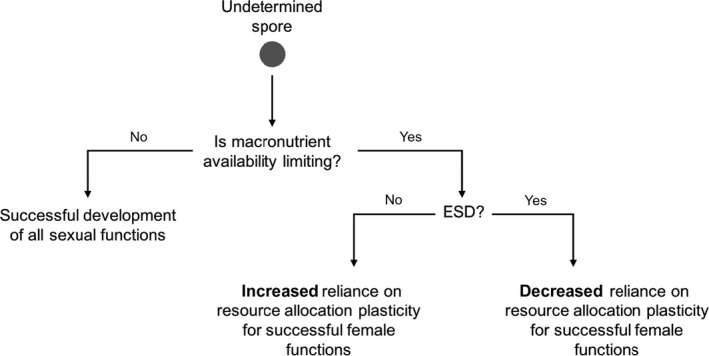
Hypothesized effect of environmental sex determination (ESD) on expected reliance on plasticity of resource allocation for ensuing sex expression, assuming female functions are more resource‐demanding than male functions in terms of absolute nutrient demands. When nutrients are not limiting, growth and reproduction can be accomplished without nutrient constraints. When resources are limiting and ESD is absent (or weak), the adaptive value of plasticity in resource allocation is predicted to be higher because some individuals in the population have not been well matched to their given environmental patch. When resources are limiting and ESD is present, there is less need for many individuals to alter their resource allocation response because their gender has been previously matched to the availability of resources in the environment. Therefore, the adaptive value of a plastic resource allocation response is diminished by prior ESD

Using the number of female eggs (one egg per archegonium; Figure 1a) per unit meristic gametophyte area as a proxy for R and total meristic gametophyte area as a proxy for V, we anticipated that nutrient limitation would result in absolute reductions in R and V for meristic gametophytes. However, the presence or absence of ESD was also expected to modulate the extent of change in R‐V (Figure 2). Populations of meristic gametophytes grown in environments where ESD occurred were predicted to exhibit relatively less change in R than those in environments where ESD did not occur, because populations that experienced ESD should already be preemptively matched to the local nutrient environment. Specifically, ESD should divert the most resource‐challenged individuals in the population away from developing the more resource‐demanding female reproductive functions. Conversely, when ESD was absent, the smallest individuals would not be preferentially eliminated from the meristic gametophyte population—resulting in relatively more variation in V—and individual meristic gametophytes would consequently be required to rely more heavily on plasticity of resource allocation to adjust their relative reproductive output.

Although preemptive sorting of individuals by ESD before sexual phenotypes arise is theoretically expected to reduce R‐V phenotypic plasticity during plant development, it is a difficult hypothesis to test in practice because contrasts of R‐V allocation with and without prior ESD are generally not possible within a single species. The *C. richardii* study system provided a rare opportunity to do so.

## 2 MATERIALS AND METHODS

### 2.1 Experimental treatments

#### 2.1.1 P limitation experiment

Two C:N:P combinations supplemented with 6 *μ*mol/L glucose (C_6_H_12_O_6_) were created. One media included P at 7.4 *μ*mol/L and the other media lacked an exogenous P source. Each nutrient treatment was replicated six times. The C, N, and P levels were selected to emulate levels often found in natural environments, with the goal of evaluating plant responses in an ecologically relevant resource context (Cleveland & Liptzin, 2007; Mathews & Chandramohanakumar, 2003; McGroddy, Daufresne, & Hedin, 2004; Sterner & Elser, 2002). The N concentration was held at 57.9 *μ*mol/L. Both media were created in a background of 1/500 altered‐basal salts media (BSM). To withhold the P normally provided in BSM as potassium phosphate (KH_2_PO_4_) without simultaneously depleting potassium (K), ammonium chloride (NH_4_Cl) and potassium nitrate (KNO_3_) were substituted for ammonium nitrate (NH_4_NO_3_). Additionally, disodium phosphate (Na_2_HPO_4_) was substituted for potassium phosphate (KH_2_PO_4_) in order to avoid changing the concentration and stoichiometry of other macro‐ and micronutrients. A 1/500 BSM treatment with N:P adjusted to 16:1, also supplemented with 6 *μ*mol/L glucose, served as a control media.

Dry, surface‐sterilized spores of *C. richardii* were sown with a sterile cotton swab onto 60‐mm Petri dishes containing solid nutrient media, resulting in culture populations between 13 and 43 individuals, and population densities between 1.6 and 6.4 plants/mm^2^. Although there was no way to precisely control individual plant spacing with this sowing technique, total plant number was included as a concomitant variable in statistical analyses. Plates were placed in a near‐airtight acrylic chamber, and the CO_2_ concentration was increased to 1,300 ppm in order to reach superelevated concentrations (>1,200 ppm), which are expected to induce responses distinctive from those observed at moderately elevated levels (400–1,200 ppm) (Kaplan et al., 2012). Levels of CO_2_ were regularly measured with a wireless NODE CO_2_ sensor (Variable Inc., Chattanooga, TN, USA). The chamber was periodically opened to replace the NODE batteries, and CO_2_ was re‐injected. The acrylic chamber was incubated in an environmental chamber (model E‐30B; Percival Scientific, Boone, USA; 30°C, photosynthetically active radiation (PAR) ~30 *μ*mol m^−2^ s^−1^, 12‐hr photoperiod) for 12 days after a 2 day induction under continuous light (30°C, PAR ~60 *μ*mol m^−2^ s^−1^). Gametophytes were grown for a total of 14 days.

#### 2.1.2 N limitation experiment

Five different C:N:P combinations supplemented with 6 *μ*mol/L glucose (C_6_H_12_O_6_) were created in a background of 1/500 diluted basal salts media (BSM; C‐Fern Web Manual, 2009). Each nutrient treatment was replicated six times. The range of N molarities was selected to emulate nutrient concentrations that may be limiting to plant growth or development. Ammonium nitrate concentrations were between 0 and 57.9 *μ*mol/L, while the potassium phosphate concentration was held constant at 7.4 *μ*mol/L.

The sowing technique described in the P limitation experiment was implemented again, resulting in culture populations between 13 and 68 individuals, and densities between 6.8 and 34.3 plants/mm^2^. Plates were located in a near‐airtight acrylic chamber environment with a CO_2_ concentration of 1,300 ppm to ensure plants were not CO_2_‐limited. Concentrations of CO_2_ were monitored in the same manner as previously described. Light conditions and duration of plant growth were also the same as previously described.

#### 2.1.3 C limitation experiment

The C:N:P combination 5:48:3, supplemented with 6 *μ*mol/L glucose, was created in a background of 1/500 diluted basal salts media (BSM) and replicated three times at both ambient and elevated CO_2_ concentrations. The elevated CO_2_ experiment was completed twice in order to increase sample size. Each block of elevated CO_2_ replicates was analyzed separately due to significant block effects. Again, the N and P molarities were selected to emulate nutrient concentrations often observed in natural environments. The ammonium nitrate concentration was 173.8 *μ*mol/L, and the potassium phosphate concentration was 22.1 *μ*mol/L.

Spores were sown as previously described. At ambient CO_2_, resulting culture populations were between 11 and 20 individuals, and densities were between 2.5 and 5 plants/mm^2^. In the two elevated CO_2_ trials, populations were between 14 and 41 individuals, and densities were between 3.2 and 9 plants/mm^2^. Plates subjected to ambient CO_2_ were incubated in an environmental chamber (model E‐30B; Percival Scientific). Plates exposed to elevated CO_2_ were placed in a near‐airtight acrylic chamber, with CO_2_ concentration increased to 1,300 ppm. Concentrations of CO_2_ were monitored in the same manner as previously described. Light conditions and duration of plant growth were also the same as previously described.

### 2.2 Gametophyte characterization

*Ceratopteris richardii* gametophytes are classified as male, female, or hermaphrodite based on the Klekowski gender classification scheme (Figure 1; Klekowski, 1969). Male gametophytes lack a lateral meristem and develop only antheridia—the site of gametophyte sperm production. Both female and hermaphroditic gametophytes exhibit a lateral meristem, with females developing archegonia (eggs) and hermaphrodites developing both archegonia and antheridia. Each gametophyte from the C, N, and P limitation experiments was categorized as an ameristic male or a meristic female/hermaphrodite. To evaluate meristic plant size and female reproductive output, all meristic gametophytes from each experimental replicate were preserved in 95% ethanol. Five meristic gametophytes selected at random from each replicate were subsequently stained with <0.1% toluidine blue and photographed with a Zeiss Primo Star light microscope equipped with an AxioCam ERc5s HD digital camera (Carl Zeiss, Göttingen, Germany). For each meristic gametophyte, three variables were scored as follows: (a) number of archegonia (eggs), (b) gametophyte area (vegetative growth (V)), and (c) the number of archegonia per unit area (mm^2^). Each mature or developing archegonium was counted toward the total number of archegonia. Meristic gametophyte area was determined from digital photographs with ImageJ as described in Hill, Germino, and Alongi (2011). The dry mass of individual gametophytes could not be obtained in order to relate individual mass to individual R, because single plants do not weigh enough to get accurate data; only pooled weights can be used to estimate average individual weights. The only methodological proxy that could be used at individual plant‐level was gametophyte area because it was available at the same time egg number was evaluated using light microscopy. The number of mature and developing archegonia per unit area per meristic gametophyte was used to quantify relative reproductive output (R).

Nutrient limitation will, by definition, reduce plant size (Sterner & Elser, 2002). In order to interpret results of nutrient limitation on *C. richardii* gametophytes, the allometric relationship between reproductive output and vegetative growth in unlimited nutrient conditions was first established. To estimate changes in R with respect to V in nutrient‐unlimited environments, the number of archegonia/unit area (R) was plotted against total gametophyte area (V) for gametophytes from the P = 1, N = 16, and elevated CO_2_ treatments. A logarithmic function was fit to the data. Deviations from that function in nutrient‐limited environments imply a change in the R‐V relationship, unexplained by decreases in size.

### 2.3 Statistical analyses

Population sex ratios in *Ceratopteris richardii* gametophyte are partly dependent on the sex pheromone *Ceratopteris* antheridiogen (A_CE_; Banks, Hickok, & Webb, 1993; Dyer, 1979; Näf, Nakanishi, & Endo, 1975; Scott & Hickok, 1987), which regulates the mating system as a function of population density (Atallah & Banks, 2015; Banks, 1999; Korpelainen, 1998; Tanaka et al., 2014; Tanurdzic & Banks, 2004; Yamane, 1998). To minimize the effects of population density as a confounding factor in sex determination, we attempted to constrain that parameter within a narrow range by regulating the number of spores initially sown; any remaining variation in spore density was randomly distributed across nutrient treatments. Additionally, total plant number was included as a concomitant variable in all ANCOVA analyses relating to the percentage of gametophytes that developed as male.

The effects of P (0, altered 1, and unaltered 1), N (0, 0.125, 0.5, 1, 5, and 16), or CO_2_ (ambient or elevated) concentration on the percentage of ameristic gametophytes was tested using an analysis of covariance (ANCOVA), with total plant number serving as the concomitant variable. Assumptions of ANCOVA (e.g., equal treatment slopes, and general linear model constraints) were met for all analyses (see Figures S1–S3). Given a significant effect of P concentration, pairwise tests were implemented to determine at which P concentrations percent ameristic males differed significantly, using Scheffe’s procedure (Scheffe, 1953). Significant pairwise differences prompted the use of an upper‐tailed, pooled variance *t* test to determine whether the treatment with P = 0 resulted in a higher percentage of ameristic males than the new P = 1 treatment.

To determine whether meristic gametophytes grown in nutrient‐limited environments (P = 0, N = 0, and ambient CO_2_) differed from their non‐nutrient‐limited counterparts (P = 1, N = 16, and elevated CO_2_, respectively) in area (mm^2^) and number of archegonia per unit area, Welch two‐tailed *t* tests were used. Sample sizes were larger than 30, allowing deferment of normality assumptions under the central limit theorem (Aho, 2013). One gametophyte in the ambient CO_2_ treatment of the CO_2_ limitation experiment was determined to be an outlier based on the 1.5 × IQR rule and was removed from the data set.

To determine whether R and V exhibit a negative relationship when nutrients are not limiting, the data were subsampled by archegonia count (i.e., all gametophytes with three, four, five, six, seven, or eight archegonia), and log(R) was plotted against V for the observations with archegonia count three through eight. Data were subsampled because we suspected that archegonia production was occurring in intervals as gametophyte size increased. An ANCOVA was used to determine whether the number of archegonia, gametophyte area, or the interaction of those two variables influenced relative reproductive output (archegonia/unit area). The slope and y‐intercept of the fitted line were estimated. All statistical analyses were performed using the R computational environment (R Core Team, 2014) with heavy reliance on the package asbio (Aho, 2014).

**Figure 3:**
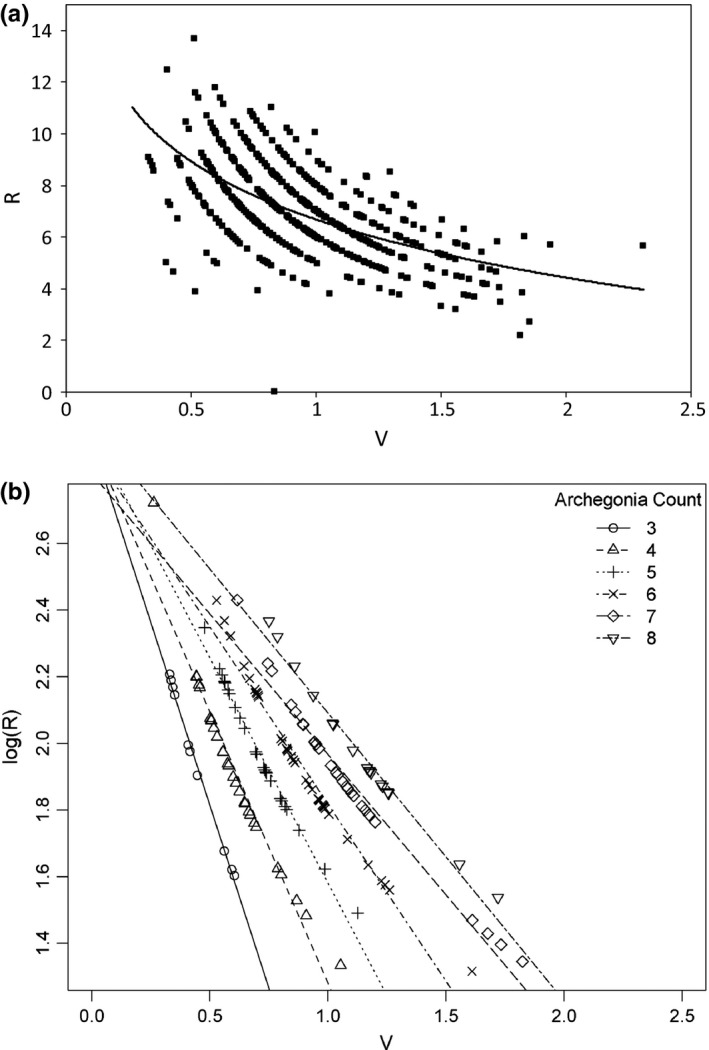
(a) Relative reproductive output (R)—measured as the number of archegonia per unit area—as a function of total gametophyte area (V), for all meristic gametophytes in the unlimited CO_2_, N, and P treatments. Normal gametophyte growth and development in non‐nutrient‐limited conditions followed a logarithmic function, depicted by the black line. As gametophyte area increased, reproductive output decreased asymptotically. Additionally, the observations separated incrementally based on whole number differences in the number of archegonia. (b) The log of R plotted as a function of V for gametophytes with archegonia counts three through eight. Based on the ANCOVA, the interaction term (V:number of archegonia) significantly influenced R (*p*‐value < 0.0001), and the R‐V relationship was negative with a slope of approximately −2.20

This study was explicitly designed to examine the effect of nutrient limitation. Nutrient limitation is defined as any nutrient level that reduces growth (i.e., V is experimentally reduced). A reduction in growth over a set time interval translates to smaller individual size. Thus, by definition, this study does not consider potential resource allocation responses where V remains unchanged or increases.

## 3 RESULTS

### 3.1 P limitation experiment

The percentage of ameristic *Ceratopteris richardii* gametophytes varied significantly between the treatments that contained P and the treatment that lacked P at α = 0.1 (Tables 1 and 2), and P limitation resulted in ESD. Total gametophyte number did not influence the percentage of ameristic males (Table 1). Based on the subsequent upper‐tailed, pooled variance *t* test, the percentage of ameristic males was significantly higher in the altered‐BSM no‐P treatment than in the altered‐BSM P = 1 treatment (*t*_10_ = 2.99, *p*‐value = 0.022). A Welch two‐tailed *t* test indicated that meristic gametophytes in treatment P = 0 were significantly smaller in area than meristic gametophytes in treatment altered‐BSM P = 1 at 14‐DAS (*t*_44.12_ = −6.02, *p*‐value < 0.0001; Table 2). Additionally, the smallest meristic gametophyte in the P = 1 treatment was 0.263 mm^2^ in area, and the smallest in the no‐P treatments was 0.240 mm^2^, representing a 0.023 mm^2^ (8.8%) decrease in area in the P = 0 treatment. The mean number of archegonia per unit area did not differ between meristic gametophytes in the P = 0 treatment and in the altered‐BSM P = 1 treatment (*t*_52.40_ = 0.28, *p*‐value = 0.78; Table 2).

**Table 1:** Summary of ANCOVA results for the effects of nutrients (CO_2_, N, or P concentration) on the percentage of ameristic male *Ceratopteris richardii* gametophytes. Total plant number served as the concomitant variable in each analysis

Experiment	Effect in model	Type II sums of squares	*df*	Mean square	*F*	*p*‐Value
CO_2_ limitation	CO_2_ concentration	48.18	2	120.30	0.15	0.87
	Total plant number	58.97	1	138.60	0.36	0.58
	Residuals	822.04	5	89.26		
N limitation	N concentration	2.8	1	2.80	0.02	0.89
	Total plant number	2,582.4	1	2,582.40	16.78	0.0003**
	Residuals	5,079.9	33	153.94		
P limitation	P concentration	1,198.7	2	599.35	3.60	0.05*
	Total plant number	249.1	1	249.10	1.50	0.24
	Residuals	2,331.8	14	166.56		

*p‐value < 0.1, **p‐value < 0.05.

**Table 2:** Summary of factor level means and standard error of the mean (SEM) for percent ameristic males in *Ceratopteris richardii* gametophyte populations, as well as meristic gametophyte area (mm^2^) and the number of eggs/unit area per meristic gametophyte in all nutrient limitation experiments (i.e., CO_2_, N, and P)

Experiment	Treatment	Percent males		Area		Eggs/unit area	
		Mean	*SEM*	Mean	*SEM*	Mean	*SEM*
CO_2_ limitation	Ambient	48.9	5.7	0.3	0.009	5.2	0.5
	Elev., #1	47.8	1.5	0.7	0.02	7.7	0.2
	Elev., #2	57.3	10.6	1.04	0.03	6.4	0.2
N limitation	0	62.6	6.3	0.9	0.03	7.2	0.3
	0.125	66.0	7.0	—	—	—	—
	0.5	70.0	4.7	—	—	—	—
	1	50.9	5.8	—	—	—	—
	5	56.9	7.2	—	—	—	—
	16	65.2	3.7	1.2	0.1	6.1	0.2
P limitation	Altered 0	71.2	3.5	0.4	0.01	7.9	0.5
	Altered 1	48.2	7.2	0.5	0.02	7.7	0.4
	1‐1/500 BSM	57.4	4.7	—	—	—	—

### 3.2 N limitation experiment

At the N concentrations tested, the percentage of ameristic gametophytes was influenced by total gametophyte number but variation in N concentration had no effect (Tables 1 and 2); ESD did not occur. A Welch two‐tailed *t* test indicated that meristic gametophytes in treatment N = 0 were significantly smaller in area at 14‐DAS than meristic gametophytes in treatment N = 16 (*t*_40.50_ = −5.23, *p*‐value < 0.0001; Table 2). The smallest meristic gametophyte in N = 16 treatment was 0.688 mm^2^ in area, and the smallest in the N = 0 treatment was 0.575 mm^2^. Thus, the smallest plant in N = 0 treatments was 0.113 mm^2^ (16.4%) smaller than the smallest gametophyte in the N = 16 treatment. Based on a Welch two‐tailed *t* test, meristic gametophytes in treatment N = 0 had more archegonia per unit area than gametophytes grown in treatment N = 16 (*t*_60.50_ = 3.22, *p*‐value = 0.002; Table 2).

### 3.3 CO_2_ limitation experiment

When grown at the same C:N:P level at two different CO_2_ levels, the percentage of ameristic *C. richardii* gametophytes was not influenced by CO_2_ concentration nor total gametophyte population, that is, ESD did not occur (Table 1). After sex determination, both elevated CO_2_ trials resulted in larger meristic gametophytes than the ambient CO_2_ treatment at 14‐DAS (*t*_70.35_ = 22.02, *p*‐value < 0.0001; *t*_45.37_ = 25.63, *p*‐value < 0.0001; Table 2). The smallest meristic gametophyte grown in the elevated CO_2_ trial 1 was 0.457 mm^2^ in area and in trial 2 was 0.408 mm^2^, whereas the smallest gametophyte grown at ambient CO_2_ was 0.249 mm^2^ in area. Therefore, the smallest meristic gametophyte at ambient CO_2_ was 0.159 mm^2^ (39.0%) smaller than the smallest gametophyte at elevated CO_2_. Additionally, both elevated CO_2_ trials resulted in meristic gametophytes with more archegonia per unit area, based on Welch two‐tailed *t* tests at α = 0.1 (*t*_14.56_ = 3.85, *p*‐value = 0.002; *t*_13.90_ = 1.81, *p*‐value = 0.09; Table 2).

### 3.4 R‐V allometric patterns

The R‐V relationship for the unlimited environments examined followed a logarithmic function (Figure 3). As plant size (V) increased, reproductive output (R) decreased, but asymptotically. The characteristic steps observed in the plots were due to incremental differences in the number of archegonia, because archegonia count was assessed only as whole number values. The relationship between V and R was negative logarithmic, with a slope of approximately −2.20 (Figure 3). Additionally, the interaction between meristic gametophyte area and the number of archegonia significantly influenced relative reproductive output (*p*‐value < 0.0001).

**Figure 4:**
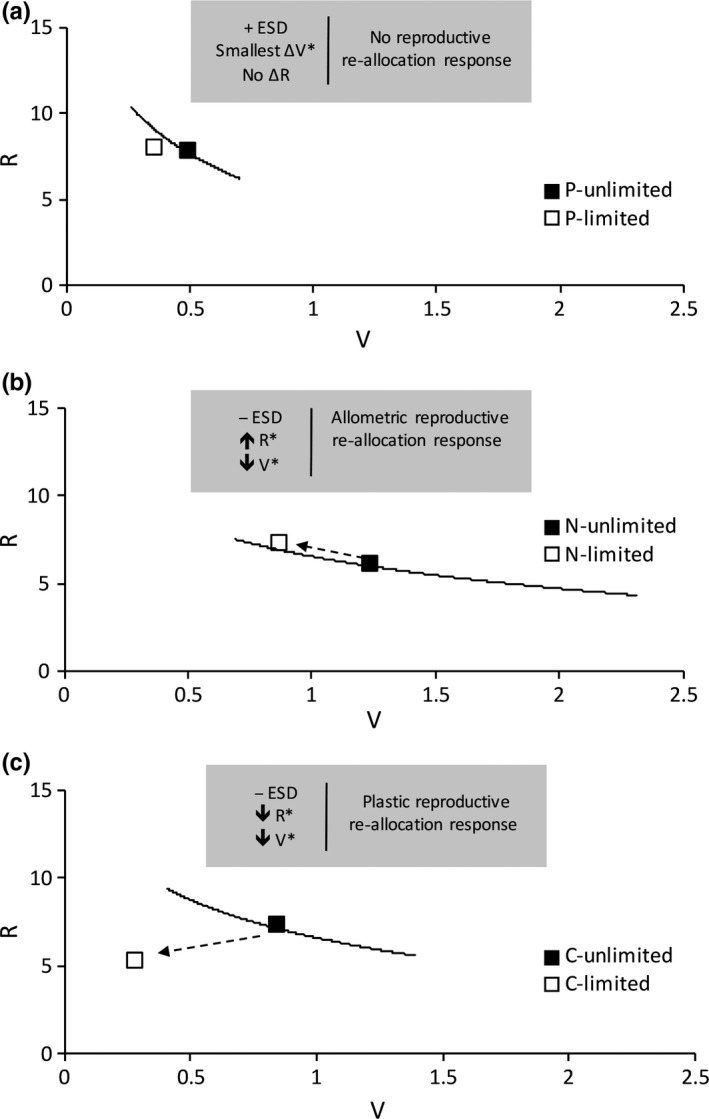
Changes in relative reproductive output (R) with respect to total gametophyte area (V), as a result of Liebig limitation. In each panel, the solid black line represents a function fit to the unlimited R‐V data for the given nutrient treatment, with the black square denoting the mean R and V values. Open squares denote the mean R and V value for the nutrient‐limited data. (a) With prior ESD, when the environment varies from unlimited to limited P, R did not significantly change even though V decreased. (b) Without prior ESD, N limitation caused a larger change in the R‐V relationship. Under N limitation, greater relative allocation to reproductive structures was due to an allometric response. In general, smaller meristic gametophytes developed more archegonia per unit area than larger gametophytes, even when N was not limiting (see Figure 3). (c) When the environment varies from non‐CO_2_‐limited (*n* = 100) to CO_2_‐limited (*n* = 13), ESD does not occur and meristic gametophytes significantly alter their reproductive allocation in response to inadequate nutrient availability. Meristic gametophytes in ambient CO_2_ are much smaller and have reduced reproductive effort when compared to gametophytes grown at elevated CO_2_. Asterisks in the gray summary boxes indicate statistical significance

To illustrate how nutrient limitation influenced the R‐V relationship of meristic gametophytes, data from the P‐, N‐, and CO_2_‐limited treatments were plotted against the characteristic R‐V allocation patterns seen in the unlimited P, N, and CO_2_ treatments, respectively (Figure 4). The mean of R from the P‐limited treatment did not significantly vary from that of the unlimited P treatment, although P‐limited gametophytes were significantly smaller in area than non‐P‐limited gametophytes (Figure 4a). The means of R and V from both the N‐ and CO_2_‐limited treatments were significantly different from their respective means in the unlimited N and CO_2_ treatments (Figure 4b,c). N limitation resulted in smaller plant size and an expected increase in R, correlated with a decrease in V (Figure 3). That is, meristic gametophytes in the N = 16 treatment were larger than those in the N = 0 treatment, but the former produced fewer archegonia per unit area than the latter (Figure 4b), whereas CO_2_ limitation caused both R and V to decrease (Figure 4c).

## 4 DISCUSSION

The extent to which nutrient availability influenced sex determination (i.e., whether ESD was expressed or not) predicted the subsequent degree of plasticity of resource allocation to female reproduction in resultant meristic *Ceratopteris richardii* gametophytes. When ESD was absent, an individual’s gender had not been preferentially matched to the local environment and plasticity of reproductive allocation at the individual level appeared to be the main mechanism used by gametophytes to developmentally adjust their phenotypes. Alternatively, when ESD was present, plasticity of resource allocation seemed to be of less importance because many individuals had already established their gender in response to cues about the prevailing environmental conditions; populations of gametophytes had matched their nutrient needs to environmental availability based on differences in resource demands by gender. Thus, nutrient limitation evoked a range of allocation strategies from a single genotype depending on whether prior ESD has occurred or not (Figures 2 and 4).

In the unlimited nutrient conditions specified, *C. richardii* gametophyte growth followed a characteristic negative, nonlinear allometric R‐V function (Figure 3), and gametophytes in the P‐limited environment did not strongly diverge from that function (Figure 4a). When N was limited or removed, or CO_2_ was limited, comparatively larger differences in area between the smallest gametophyte in the nutrient‐unlimited environments and the smallest gametophyte in the nutrient‐limited environments were evident. However, that same strong effect of a limiting nutrient reducing growth was not observed when P was removed from the environment. Although plant area was reduced in the P‐limited treatment, the difference in area between the smallest gametophyte grown without P and the smallest grown with P was less than that in the other two limitation experiments in absolute and relative terms. Therefore, failure to significantly shift the R‐V relationship as a result of P limitation was specifically due to a relatively small change in V among meristic gametophytes (Figure 4a).

**Figure 5:**
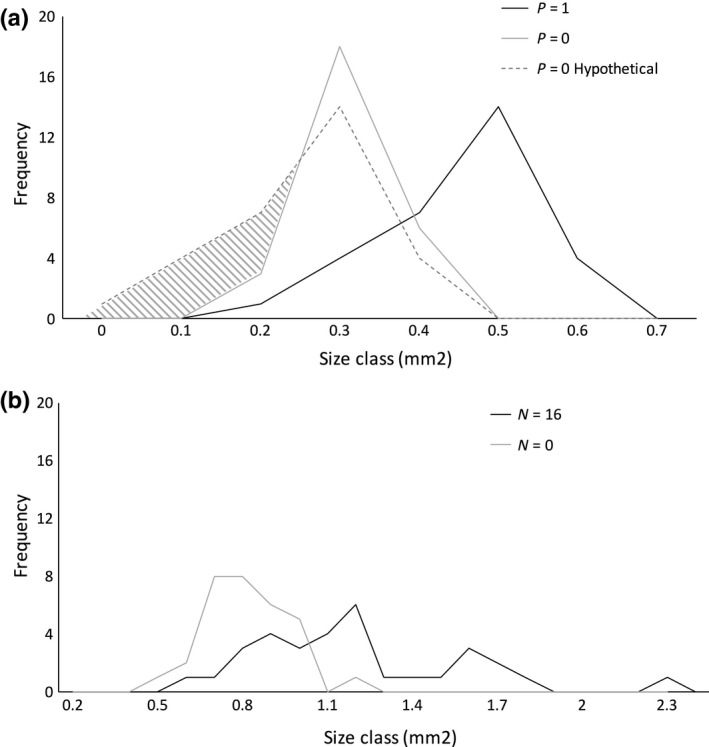
Size class frequency distributions in P limitation (a) and N limitation (b) experiments. Although both the P‐unlimited (black line) and the P‐limited (solid gray line) data resulted in left‐skewed distributions (a), the size difference between the smallest gametophyte in the P‐limited environment and the smallest gametophyte in the unlimited P environment was insufficient (8.8%) to prompt a substantial change in the R‐V relationship. If gametophyte size had decreased proportionately as a result of P limitation (dashed line), a size difference between the limited and unlimited environments comparable to that observed when N was limited (gray hashed area) would be expected. However, as a result of environmental sex determination (ESD), gametophytes that would have developed into the smallest of females appear to have developed into males instead. In the N limitation experiment (b), data from both the N‐unlimited and the N‐limited environments resulted in distributions that were skewed right; the mean gametophyte size was larger than the most prevalent gametophyte size. The smallest meristic gametophyte in the limited environment (solid gray line) was considerably smaller (16.4%) than the smallest gametophytes in the unlimited environment (black line). Therefore, the lack of ESD resulted in a larger size effect when N was removed compared to when P was removed

Under P limitation, ESD increased the proportion of undetermined gametophytes that developed as male, thus shifting them out of the future population of meristic phenotypes. That suggests that at least some individuals emerging from spore coats in those cultured populations were P‐limited, and sex expression among individuals was partitioned so that gametophytes in the population that would have become the smallest females instead developed into males (Figure 5a) in a manner consistent with ESD theory (Charnov & Bull, 1977). That shift appeared to constrain the decrease in V of remaining meristic gametophytes, reducing the need for plasticity of R and resulting in no meaningful change in the R‐V relationship. Consequently, resultant meristic gametophytes were better matched to the local nutrient environment and their relative reproductive output was not affected by P availability; the observed plasticity of reproductive allocation owing to variation in the nutrient environment decreased. It was impossible to explicitly prove the inference that spores that became males due to ESD would have otherwise became small females with modified reproductive allocation. However, the observed increase in the percentage of males—the smaller sex in this dimorphic species—due to ESD when P was removed and the increased frequency of small females in the N‐limited treatment where ESD did not occur (Figure 5b) are both consistent with our hypothesis.

The strain of *C. richardii* gametophytes used in this work is sensitive to the sex pheromone *Ceratopteris* antheridiogen (A_CE_) at the time of germination. Therefore, it is possible that nutrient availability altered the relative rate of secretion and/or A_CE_ sensitivity of developing gametophytes, resulting in an A_CE_‐mediated change in the proportion of gametophytes that developed as male (Banks et al., 1993; Dyer, 1979; Näf et al., 1975; Scott & Hickok, 1987). However, under that assumption, abiotic environmental conditions were still providing a key signal about habitat patch quality that was used for sex determination. Other inbred genetic strains of *C. richardii* are less sensitive to A_CE_ (Scott & Hickok, 1987). It would be interesting to determine whether A_CE_‐insensitive lines respond to nutrient limitation in different ways compared to the genotype studied here. Regardless, our study did not attempt to explain the proximate mechanisms by which nutrients regulate sex determination; instead, we used our ability to alter the sex ratio in populations of fully inbred *C. richardii* gametophytes to provide a rare empirical test of hypotheses about the evolutionary importance of ESD on the expression of phenotypic plasticity in resource allocation.

Significant changes in R allocation appeared to be unavoidable in the absence of ESD (Figure 4b,c). Specific patterns of resource re‐allocation to different structures can be either (a) a response to a proportional or ratio‐driven process, because environmental resource pools are finite at any given time (Weiner, 2004), or (b) a consequence of plant size, where allometric patterns evolve in response to selection pressures and constraints (Müller, Schmid, & Weiner, 2000). In this study system, meristic gametophytes were previously shown to be the more N‐rich sex in both absolute and relative terms (Goodnoe & Hill, 2016). Yet, even when N was completely removed from the nutrient environment, the percentage of male gametophytes did not increase, suggesting that meristem formation in *C. richardii* gametophytes required that element in very small absolute amounts.

Although elimination of N from the environment subsequently reduced growth and size of meristic gametophytes at 14‐DAS, those gametophytes actually increased allocation of nutrients to reproductive structures compared to meristic gametophytes in the highest N treatment (Figure 4c). Gametophytes in the N‐limited treatment made a trade‐off between investment in vegetative and reproductive growth. Although originally unanticipated, an increase in R was ultimately expected when considered in the context of allometric changes in allocation observed during normal meristic gametophyte growth in nonlimiting nutrient conditions (Figure 3). In *C. richardii*, reproductive allocation to egg production was initially high and gradually decreased as meristic gametophyte size increased (Figure 3). Therefore, although the R‐V relationship of meristic gametophytes changed when gametophytes were N‐limited, that change was largely attributable to a shift in position along the normal R‐V growth function owing to small size (Figures 3 and 5b); it was an allometric response resulting in apparent plasticity (Bishop, Spigler, & Ashman, 2010; Delerue et al., 2013). We would expect that if gametophytes in the N‐limited treatment could escape nutrient limitation and continue growth beyond 14‐DAS, they would decrease their relative reproductive output as a correlated response to increasing individual plant size, and thus behave like the gametophytes in the non‐N‐limited treatment in terms of allocation strategy.

Increasing the concentration of CO_2_ also did not elicit ESD (Table 1), suggesting that sex determination in the population subjected to CO_2_ limitation was established based on the concentration of antheridiogen sex pheromone alone. A consistent sex ratio between ambient and elevated CO_2_ treatments indicates that the concentration of CO_2_ in the ambient environment was sufficient to initiate formation of an active meristem. However, C limitation subsequently resulted in a disproportionate decrease in R relative to the decrease in V of meristic *C. richardii* gametophytes, which was accomplished by an obvious phenotypic departure from the normal R‐V growth function (Figure 4c). Smaller gametophytes in ambient CO_2_ exhibited reduced reproductive allocation unexplained by a change in size, thus resulting in a truly plastic response. This response is similar to sex allocation plasticity observed in angiosperm sporophytes, where hermaphroditic individuals greatly differ in their investment in female sexual function as a result of nutrient availability variation (Bishop et al., 2010; Dorken & Mitchard, 2008).

Elevated environmental CO_2_ concentrations have the potential to critically alter plant communities via changes in strategies for allocation to growth and reproduction (Wang, Taub, & Jablonski, 2015). In the present study, experimental manipulation of CO_2_ resulted in the largest discrepancy in reproductive output between gametophytes grown in limited and unlimited conditions. Furthermore, vegetative growth was greatly influenced by variation in CO_2_ concentration; meristic gametophytes grown at elevated CO_2_ were on average three times larger than those grown at ambient CO_2_, suggesting *C. richardii* gametophytes were C‐limited when grown in vitro at ambient CO_2_ concentrations even when a low level of exogenous glucose was available. Growth at elevated CO_2_ also allowed meristic gametophytes to allocate a greater proportion of total resources to reproductive development (Figure 4c). Ong, Koh, and Wee (1998) also observed that size and growth rate of *Pyrrosia piloselloides* gametophytes increased as a result of growth under elevated CO_2_ conditions. It is possible that CO_2_ limitation in gametophyte ecology has broad importance, functioning to significantly alter the R‐V relationship, resulting in novel phenotypes that are far from the normal allometric function plants follow when nutrients are not limiting.

Because C is the most abundant element in meristic *C. richardii* gametophytes, comprising 40%–60% of gametophyte dry biomass, the probability of eliciting true plasticity of reproductive allocation based on natural environmental variation in resource availability might correlate with the absolute demand for that resource. The CO_2_‐limited gametophytes appeared to prioritize investment in a certain amount of meristematic growth in order to accomplish formation of at least one mature archegonium and then reduced any further investment in vegetative growth, whereas gametophytes grown in elevated CO_2_ continued growth well after the minimum size for archegonial development had been reached. In order to successfully accomplish sporophyte formation, meristic gametophytes must reach a critical size (Sakamaki & Ino, 1999). The onset of sexual maturity at a small size—as occurred in both the ambient CO_2_ and N‐limited experimental conditions—may result in a shorter lifespan and accelerated, though unsuccessful, sporophyte formation, and consequently decreased individual fitness (Greer & McCarthy, 1999).

## 5 CONCLUSIONS

The current work exemplifies the expected negative relationship between R and V (Figure 3) that has previously proven difficult to demonstrate in practice, by empirically testing the theory of allocation trade‐offs in individuals within a genetically uniform population by means of a simple, land plant model system. We were able to discern the effects of labile sex determination on subsequent plasticity of nutrient allocation to reproductive output by creating environments where populations of individuals did and did not exhibit ESD (Figure 4). In this case, ESD acted as an avoidance strategy by reducing the probability of low‐quality females developing in a population. The available evidence suggests those females may not have reached the minimum size threshold for reproduction through female function (Figure 5). Thus, in *C. richardii* gametophytes, population‐level ESD allowed individuals to avoid the need for large, potentially futile adjustments in future resource allocation because of decreased size resulting from nutrient limitation by preemptively shifting the population sex ratio toward males. To our knowledge, there exists no other work that explicitly examines the effects of variation in ESD on resultant whole‐organism female resource allocation within a plant species.

## CONFLICT OF INTEREST

None declared.

## AUTHOR CONTRIBUTIONS

T.G. and J.H. contributed to design and implementation of the research project, as well as manuscript development and editing; T.G. collected and analyzed data, and authored the manuscript, figures, and tables.
