# Supplementary material for: Plasticity of female reproductive resource allocation depends on the presence or absence of prior environmental sex determination in Ceratopteris richardii
Source: Ecol Evol. 2018 May 20;8(12):6133–43. doi: 10.1002/ece3.4159 (PMC6024121; doi:10.1002/ece3.4159)
Supplement: Supplementary file 1 [file ECE3-8-6133-s001.pdf]

Supplementary Material

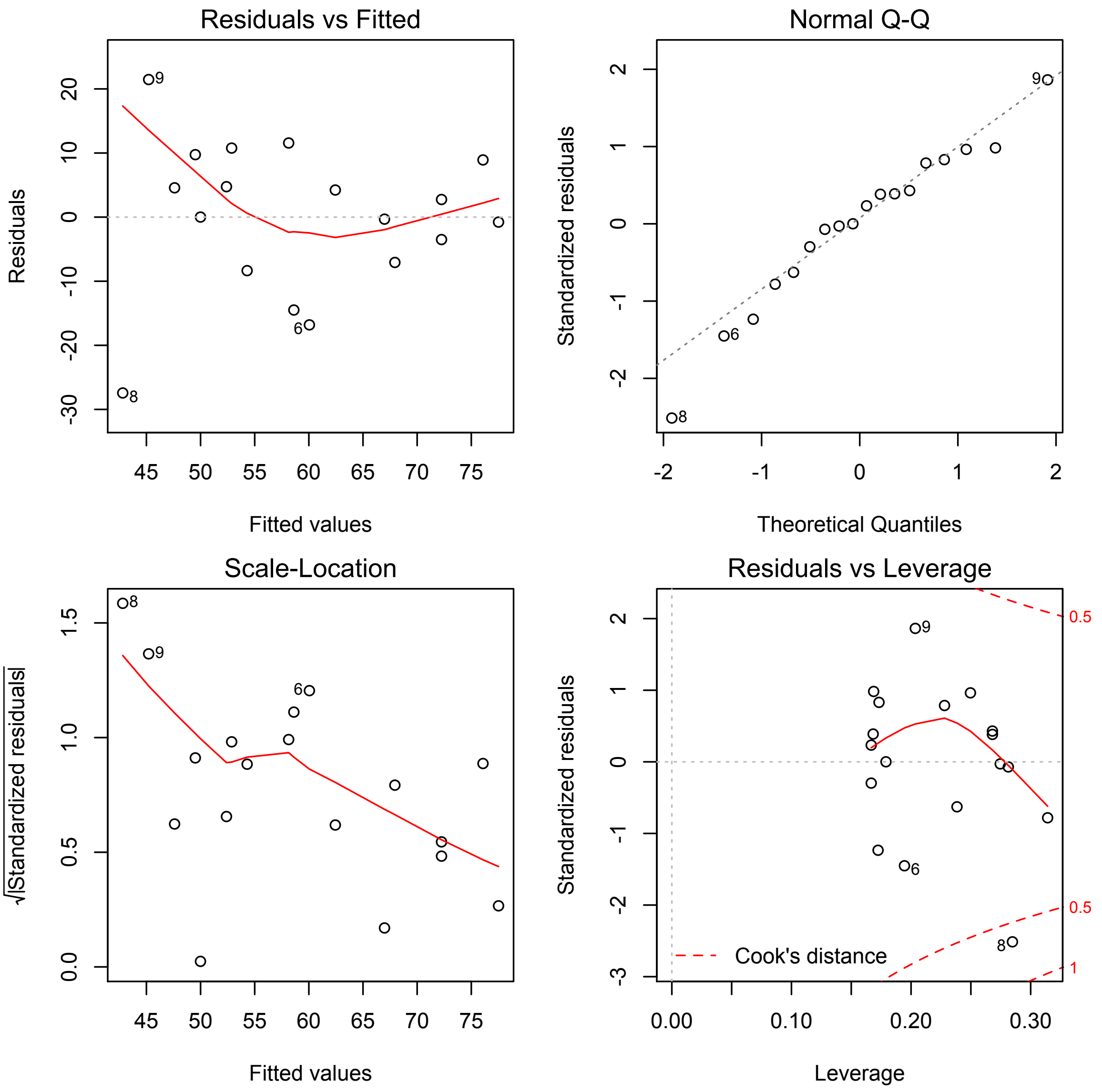

Figure S1. Default diagnostic plots produced for the P-limitation experiment. Individual observations are assumed independent because there is no obvious trend in the residuals vs. fitted plot. The linear relationship in the normal Q-Q plot and the Shapiro-Wilk normality test (*P*-value = 0.73) suggest that the data is normally distributed. Because there is not a shot-gun pattern in either the residuals vs. fitted plot or the scale-location plot homoscedasticity can be assumed. Based on the residuals vs. leverage plot, observation number 8 may be an outlier.

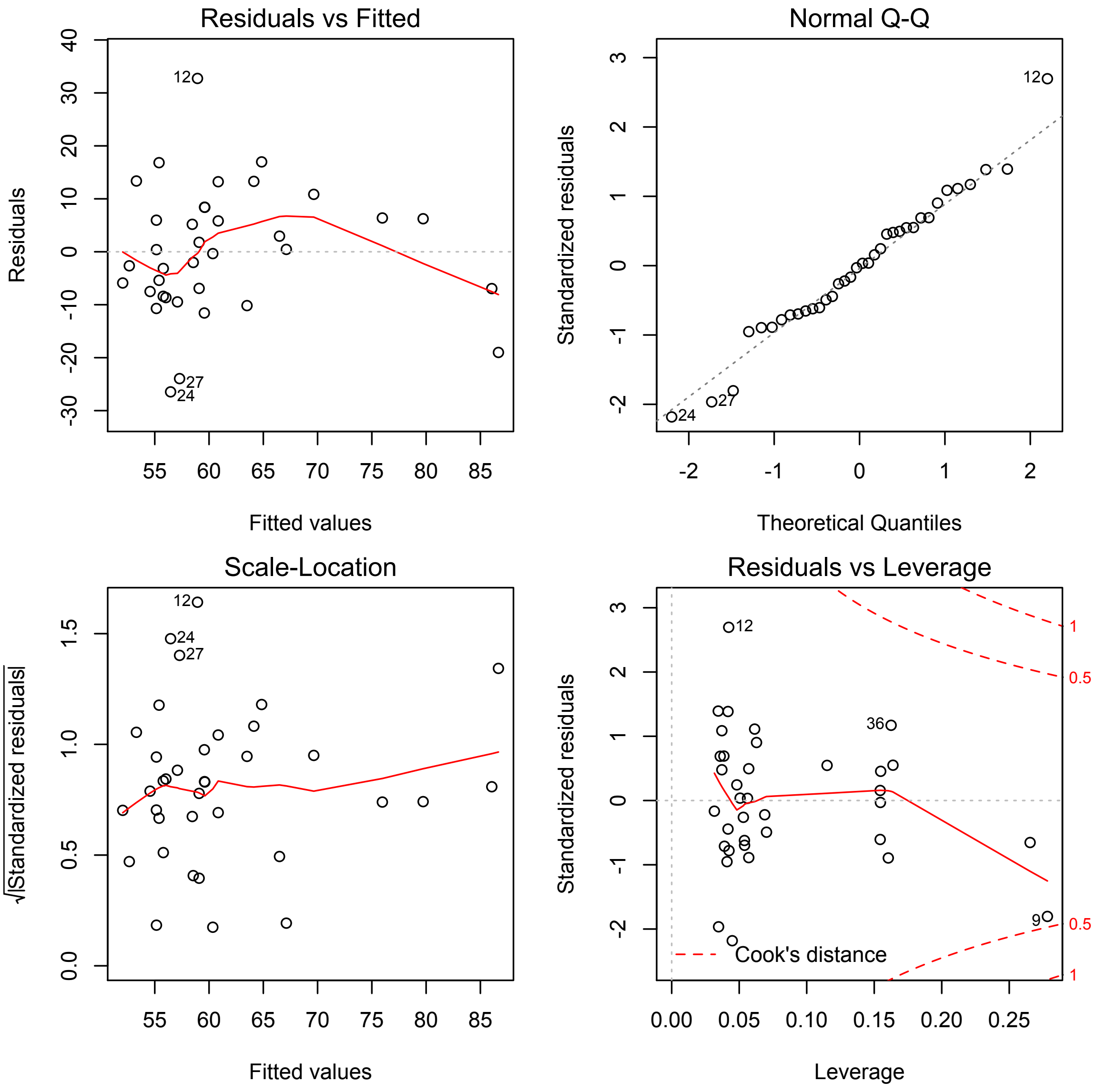

Figure S2. Default diagnostic plots produced for the N-limitation experiment. Individual observations are most likely independent because there is no obvious trend in the residuals vs. fitted plot. The linear relationship in the normal Q-Q plot and the Shapiro-Wilk normality test (*P*-value = 0.75) suggest that the data is normally distributed. Since there is not a shot-gun pattern in either the residuals vs. fitted plot or the scale-location plot homoscedasticity can be assumed.

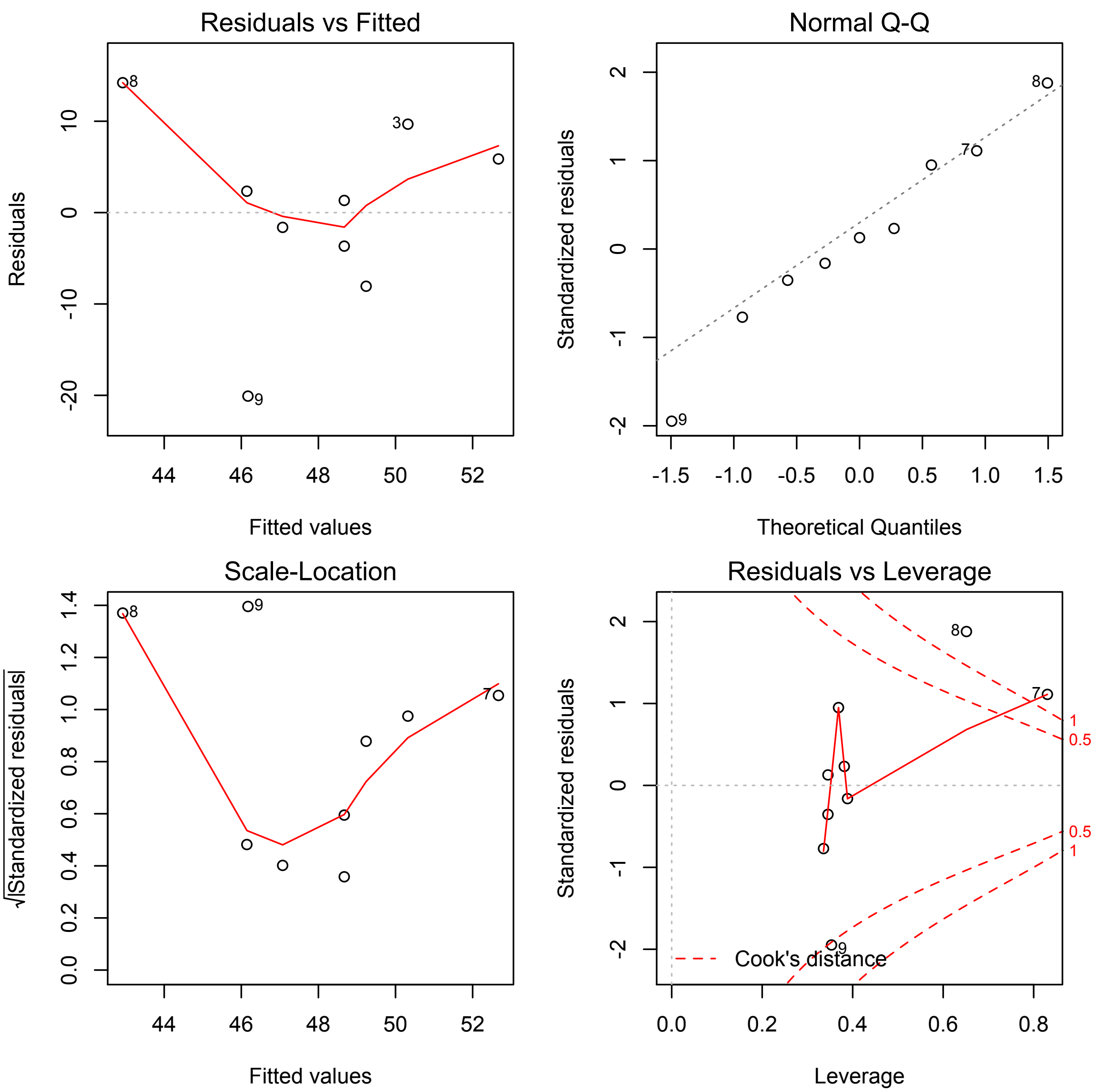

Figure S3. Default diagnostic plots produced for the C-limitation experiment. Individual observations are assumed independent because there is no trend in the residuals vs. fitted plot. The linear relationship in the normal Q-Q plot and the Shapiro-Wilk normality test (P-*value = 0.88*) suggest that the data is normally distributed. Since there is not a shot-gun pattern in either the residuals vs. fitted plot or the scale-location plot, homoscedasticity can be assumed. Based on the residuals vs. leverage plot, observations number 7, 8, and 9 may be outliers.

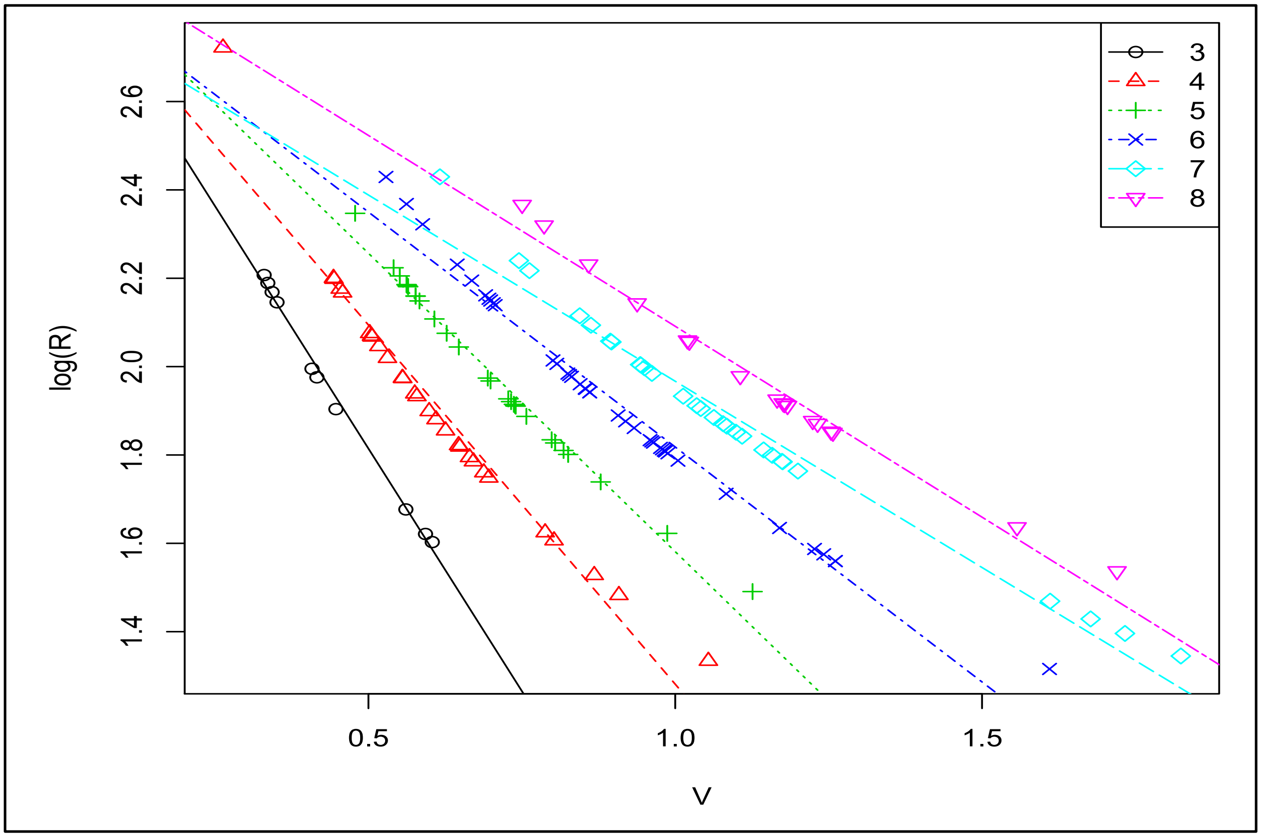

Figure S4. The log of R (relative reproductive output, measured as the number of archegonia per unit area per meristic gametophytes) plotted as a function of V (vegetative output, measured as gametophyte area) for meristic gametophytes with archegonia counts three through eight. Based on the ANCOVA, the interaction term (V:number of archegonia) significantly influenced R (*P*-value <0.0001) and the R-V relationship is negative with a slope of approximately -2.20.
